# Effect of a prenatal lifestyle intervention on physical activity level in late pregnancy and the first year postpartum

**DOI:** 10.1371/journal.pone.0188102

**Published:** 2017-11-27

**Authors:** Birgitte Sanda, Ingvild Vistad, Linda Reme Sagedal, Lene Annette Hagen Haakstad, Hilde Lohne-Seiler, Monica Klungland Torstveit

**Affiliations:** 1 Faculty of Health and Sport Sciences, University of Agder, Kristiansand, Norway; 2 Department of Obstetrics and Gynecology, Southern Norway Hospital Trust, Kristiansand, Norway; 3 Norwegian School of Sports Science, Oslo, Norway; TNO, NETHERLANDS

## Abstract

**Background:**

Despite documented health benefits for mother and baby, physical activity (PA)-level tends to decline in pregnancy. Overweight/obese and physically inactive women are two selected groups at increased risk of pregnancy complications. Thus, efficient strategies to maintain or increase PA-level in pregnancy and the postpartum period, especially among these women, are warranted. This secondary analysis examined the effect of a prenatal lifestyle-intervention on PA-level in late pregnancy and the first year postpartum, with subanalysis on initially physically active versus inactive and normal-weight versus overweight/obese women.

**Method:**

The Norwegian Fit for Delivery (NFFD) randomized controlled trial included healthy primiparous women with singleton pregnancies and body mass index (BMI) ≥19 kg/m^2^ assigned to an intervention group, n = 303 (twice weekly group-exercises and dietary counseling) or a control group, n = 303 (standard prenatal care). The International Physical Activity Questionnaire short-form was used to assess PA-levels at inclusion (mean gestational week (GW) 16), GW 36, and six and 12 months postpartum.

**Results:**

At GW 36, a positive intervention-effect with a significant between-group difference in total PA-level compared to time of inclusion was found for the total group (530 MET-min/week, *p* = 0.001) and the subgroups of normal-weight (533 MET-min/week, *p* = 0.003) and initially active women (717 MET-min/week, p<0.001). Intervention-effect was dependent on exercise-adherence among overweight/obese and inactive women. Compared to time of inclusion, the intervention groups maintained total PA-level at GW 36, while total PA-level decreased in the control groups. The PA-levels increased postpartum, but with no significant differences between the randomization groups.

**Conclusion:**

The NFFD prenatal combined lifestyle intervention had a significant effect on TPA-level in late pregnancy among women entering pregnancy normal-weight or physically active, thereby preventing the downward trend typically seen during pregnancy. Intervention-effect among overweight/obese and physically inactive women was, however, dependent on exercise-adherence. Long-term intervention-effect was not observed in the postpartum period.

## Introduction

Physical activity is currently considered safe and beneficial in healthy pregnancies, and pregnant women are recommended to be physically active for at least 150 minutes per week at moderate intensity [1–3]. Physical activity is associated with decreased risk of developing gestational diabetes [4–6], pelvic girdle pain [7], hypertensive disorders [4, 8] and excessive gestational weight gain [6, 9, 10]. Prenatal physical activity may also increase psychological wellbeing [11], maintain physical fitness [12] and improve weight management in the postpartum period [13, 14]. A large proportion of the general population is physically inactive [15, 16] and during pregnancy, physical activity level tends to decline even further [1, 17–19].

Furthermore, studies have shown that physical inactivity in pregnancy is more prevalent among overweight and obese women [18, 20]. High pre-pregnancy body mass index (BMI) is associated with increased risk of adverse obstetrical outcomes [21], such as gestational diabetes [22], preeclampsia [23] and caesarean delivery [24]. Consequently, overweight or obese women may especially benefit from increasing their physical activity level in pregnancy. However, little is known regarding the effect of lifestyle interventions on physical activity level when comparing normal weight and overweight/obese pregnant women.

A higher pre-pregnancy physical activity level has been shown to be a predictor of physical activity level during pregnancy [20, 25, 26], but whether lifestyle interventions in pregnancy influence physical activity level differently in initially physically inactive versus physically active pregnant women is not known.

Randomized controlled trials involving a combined lifestyle intervention [10, 27–31] or a physical activity intervention alone [5, 12, 32–34] show varying effect on physical activity level in pregnancy, despite proper study size and design. Further, many of the studies are designed only for overweight or obese women [5, 10, 27, 28, 30, 34] and few involve the postpartum period [10, 30].

The Norwegian Fit for Delivery (NFFD) trial was originally designed to evaluate whether an antenatal lifestyle intervention combining physical activity and dietary elements would result in measurable health benefits for both mother and baby [35]. We have previously reported that the NFFD intervention resulted in significantly improved self-reported total physical activity level [14] and diet [36] in late pregnancy, as well as a significant reduction in gestational weight gain of 1.3 kg from pre-pregnancy to term in the intervention group compared to the control group [9].

In this secondary analysis we aimed to investigate more comprehensively the effect of the NFFD lifestyle-intervention on physical activity, by assessing the reported level of walking, physical activity at moderate and vigorous intensities, as well as total physical activity. In addition, we aimed to evaluate the proportion of women meeting physical activity recommendations, both in late pregnancy and the first year postpartum. Further, we wanted to explore whether the effect of the intervention differed based on participants’ physical activity level (inactive vs. active) or BMI category (normal weight versus overweight/obese) at trial inclusion, or based on adherence to the supervised exercise classes provided as part of the intervention.

## Method

### Design

The NFFD study was a prenatal combined lifestyle intervention evaluated in a randomized, blinded, controlled trial. The study protocol has been previously published [35].

### Ethics

The Norwegian Regional Committee for Medical Research Ethics South-East-C approved the trial and modifications (REK reference 2009/429) and the trial was registered at ClinicalTrials.gov with ID NCT01001689.

### Subjects and sample size

**Fig 1:**
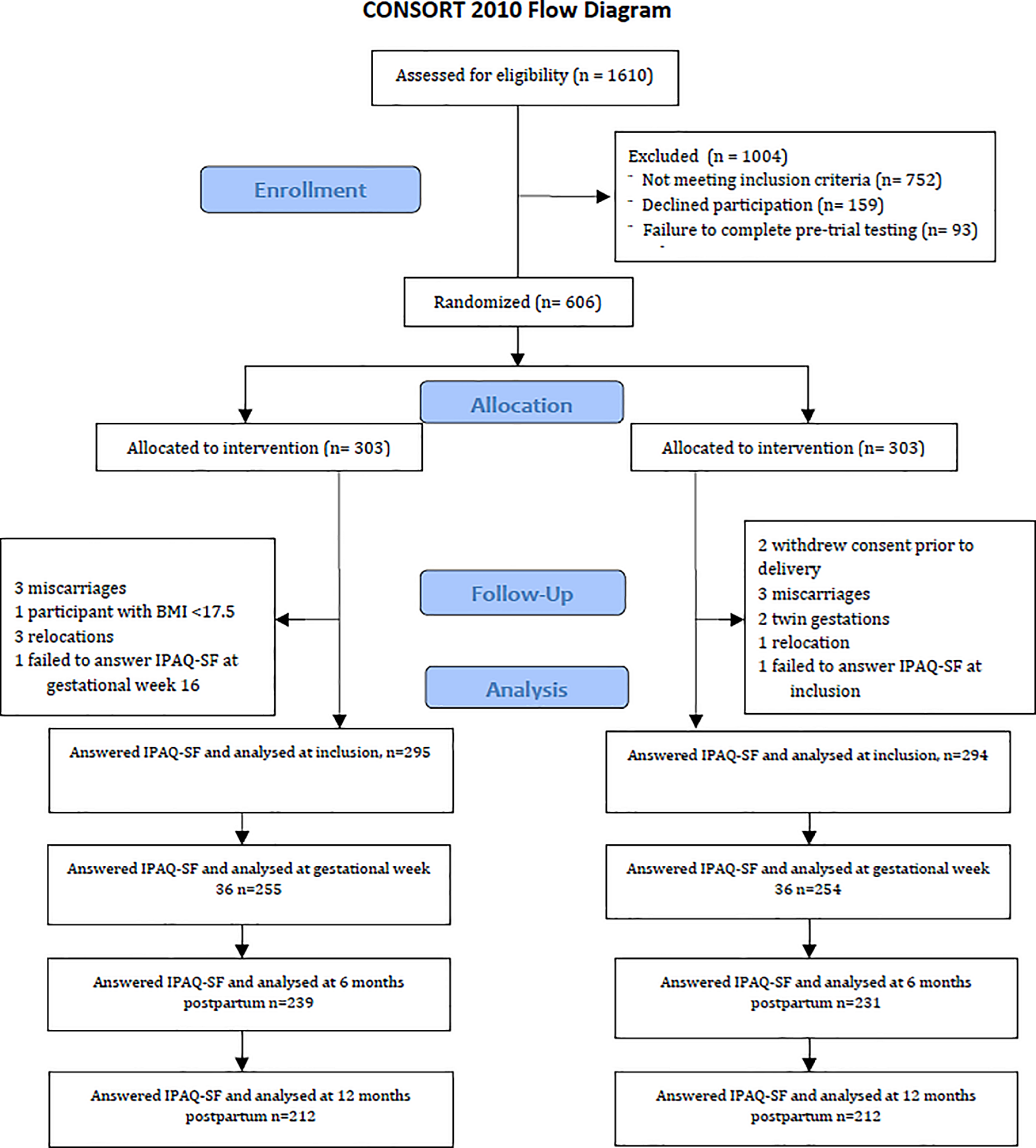
Consort diagram showing the flow of participants throughout the study.

The NFFD trial included 606 nulliparous women, comprising both normal weight and overweight or obese participants. Participants were recruited by midwives from eight healthcare clinics in the southern part of Norway, encompassing both cities and rural areas, between September 2009 and February 2013. Eligible participants were healthy nulliparous women who were literate in Norwegian or English, ≥ 18 years old, with a singleton pregnancy of ≤ 20 weeks of gestation and a pre-pregnancy BMI ≥ 19 kg/m^2^. After providing informed consent and completing initial blood tests and questionnaires, the participants were individually randomized to either the intervention (n = 303) or the control group (n = 303). Surveys included questions on physical activity level and dietary habits and were answered at time of inclusion, gestational week 36, as well as six- and 12 months postpartum. The details and rationale behind the NFFD trial sample size and randomization have been previously published [9, 35]. Fig 1 outlines flow of participants in this secondary analysis of the intervention´s effect on physical activity level.

### Intervention

From time of randomization (mean gestational week 17.6±2.6, range 7.7 to 24.1) until delivery (mean gestational week 39.9±1.8, range 31.1 to 42.6), the intervention group had access to twice-weekly group exercise classes, each lasting 60 minutes. Exercise classes were offered at five different fitness centers with an identical exercise program tailored for pregnancy, and the instructors were quality controlled by the NFFD team. The classes consisted of ten minutes of warm up, 40 minutes of cardiovascular- and strength exercises at moderate intensity and ten minutes of stretching. The strength exercises included series of bodyweight squats, standing hip flexion to extension without external resistance, single leg curl without external resistance, push-ups, chest press, triceps dip, seated/standing cable row with resistance band, reverse flyes, shoulder raise and side lateral raise with resistance band, quadruped arm leg raise, quadruped hip extension, transverse abdominis isolations on a wall, pelvic tilt exercise, plank, side plank with rotation and pelvic floor exercises. The intensity of the exercise was self-monitored using Borg’s scale for perceived exertion [37], which is recommended in pregnancy because of variations in maternal heart-rate responses to exercise [1]. For moderate intensity exercise, self-perceived exertion was set to 12–14 on the 6–20 scale [37]. The instructors recorded group exercise attendance. For practical and economical reasons the group exercise classes were limited to twice weekly. In addition, the participants were encouraged to do cardio exercise for at least 30 minutes at moderate intensity at least three times a week, in accordance with contemporary physical activity recommendations [38].

The dietary component consisted of ten recommendations designed by the NFFD team to increase awareness of food choices and meal regularity, decrease food portion sizes and intake of snacks, and increase intake of water, fruits and vegetables [35]. Participants answered a food frequency questionnaire, and adherence to the dietary recommendations was assessed using a diet score, which has been previously published [39]. The lifestyle recommendations were reinforced by pamphlets mailed to the participants, access to a password-protected NFFD website, two telephone consultations on dietary behaviour, invitation to one evening cooking class, as well as one evening meeting which provided information about the NFFD trial and the benefits of a healthy diet and regular physical activity in pregnancy.

### Control group

Participants in the control group received standard Norwegian prenatal care, comprising eight routine prenatal contacts free of charge, provided through alternating visits with midwives and doctors including one second-trimester ultrasound examination, with additional care as needed. In Norway, all pregnant women receive a booklet with advice on prenatal nutrition and physical activity, including recommendations for weight gain based on IOM guidelines [40].

### Physical activity assessment

Physical activity level was assessed by the International Physical Activity Questionnaire short-form (IPAQ-SF) [41, 42]. The IPAQ-SF quantifies physical activity during the last seven days divided into four categories: vigorous intensity physical activity (VPA), moderate intensity physical activity (MPA), walking and sitting. For each category, the dimensions of frequency and duration of physical activity are assessed. Participants in both the intervention- and the control group answered IPAQ-SF either electronically (>90% of participants, in Norwegian) or in print (in English or Norwegian), at inclusion and gestational week 36, as well as six- and 12 months postpartum. Responses were scored according to established methods (www.ipaq.ki.se). For each physical activity category (walking, MPA and VPA), the reported frequency (days/week) was multiplied by reported average duration (minutes/day) and by the corresponding IPAQ algorithm (3.3 for walking, 4.0 for MPA and 8.0 for VPA) to calculate the corresponding metabolic equivalent of task (MET) in minutes/week. One MET is equivalent to the energy expenditure at rest (1 MET = 3.5 ml O_2_ x kg^-1^ x min^-1^). Total physical activity was calculated by adding walking + MPA + VPA.

### Other measurements

Maternal sociodemographic variables such as age and gestational week at inclusion, pre-pregnancy weight and BMI, educational level, occupation, household income and smoking status were obtained from the questionnaire answered at inclusion (mean gestational week 16.1±2.5), thereafter verified and supplemented by data collected at first follow-up at gestational week 30. Citizenship was obtained from hospital records.

### Sub-analysis

In the sub-analysis, the participants were stratified as inactive (not meeting recommended physical activity level at time of inclusion, < 600 MET-minutes/week of total physical activity) or active (meeting recommended physical activity level at time of inclusion, ≥ 600 MET-minutes/week of total physical activity), as well as into normal weight (pre-pregnancy BMI 19–24.9 kg/m^2^) or overweight/obese (pre-pregnancy BMI ≥25 kg/m^2^).

Participants were classified as adherent to the group-exercise intervention if they attended ≥ 15 (median value) out of approximately 40 possible group-exercises classes.

#### Physical activity recommendations

Several authorities recommend pregnant women to engage in 150 minutes of at least MPA/week [2, 3] or 20-to-30 minutes of MPA/day on most or all days of the week [1]. One hundred and fifty minutes of MPA equals 600 MET-minutes of MPA according to the IPAQ-protocol. Calculating proportions meeting physical activity recommendations at a given measure point was based on </≥ 600 MET-minutes/week of total physical activity level.

### Statistical analysis

Analyses followed the intention-to-treat principle. Descriptive statistics are presented as mean (standard deviation (SD)), median (inter-quartile-range (IQR)) or proportions, as appropriate. Differences between groups at inclusion were examined using chi-square test for categorical variables and two-sided independent sample t-test for continuous variables.

Test for normality was performed using Shapiro-Wilk test. Data was normalized by a log transformation of physical activity level measured at inclusion and gestational week 36, as well as six and 12 months postpartum when appropriate.

A mixed repeated measures Analysis of Variance (ANOVA) was conducted to assess the impact of the intervention on the participant’s physical activity level across four measurement points. The analysis excluded cases pairwise. Within subjects factor was physical activity level (total physical activity score at inclusion, gestational week 36, six months- and 12 months postpartum), and between subjects factor was randomization (intervention versus control). The following variables were included in the adjusted analysis: age and gestational week at inclusion, pre-pregnancy BMI (kg/m^2^), smoking status (yes/no), educational level (</≥4 years of college/university) and household income (</≥400 000 NOK).

When assessing possible changes in physical activity level at different measure points compared to time of inclusion, Wilcoxon Signed Ranked Test and Man Whitney U Test were used due to skewed data. Analysis included only data from responders at the different measure points.

Comparison of proportions meeting physical activity recommendations was performed using MedCalc for Windows, version 12.7.7.0 (MedCalc Software, Ostend, Belgium).

Data analysis was performed using SPSS for IBM statistical software package version 22.0 and 23.0 (IBM Corporation, Armonk, NY, USA). A *p*-value of ≤ 0.05 was considered statistically significant.

## Results

There were no significant differences in maternal characteristics between the intervention- and the control groups at inclusion (Table 1). Mean gestational age at delivery was 39.9 weeks (SD 2.0) for the control group and 39.9 (SD 1.8) for the intervention group (*p* = 0.89). As shown in Tables 2–4, no significant differences in physical activity level between the intervention and the control group were seen at inclusion for either the total sample, the subgroups of physically active or inactive women or when stratified into BMI categories.

**Table 1:** Baseline characteristics of participants for the total group (n = 598), the intervention group (n = 295) and the control group (n = 294).

	Total	Intervention group	Control group	
Variable	n = 589	n = 295	n = 294	
	Mean (SD) / Median (IQR)				*p*-value
Age at inclusion (years)	28.0 (4.35)	27.9 (4.24)	28.1 (4.46)	0.50
Gestational week at inclusion	16.1 (2.45)	16.1 (2.47)	16.1 (2.44)	0.75
Prepregnancy weight (kg)	65 (59–73)	65 (59–74)	64 (59–73)	0.53
Prepregnancy BMI (kg/m^2^)	22.7 (21.0–25.4)	22.7 (21.0–25.7)	22.7 (20.9–25.0)	0.42
		**n (%)**		
Educational level < 4 years college/university ≥4 years college/university	192 (32.7) 209 (35.5)	104 (35.4) 96 (32.5)	88 (29.9) 113 (38.4)	0.19
Occupation Employed outside home Long-term sick leave	496 (84.2) 11 (1.9)	240 (81.4) 6 (2.0)	256 (87.1) 5 (1.7)	0.055
Household income (NOK) ≤ 400,000 401,000–700,00 > 700,000 Don´t want to answer	183 (31.2) 163 (27.7) 202 (34.5) 39 (6.6)	95 (32.2) 82 (27.8) 101 (34.2) 17 (5.8)	88 (30.1) 81 (27.7) 101 (34.6) 22 (7.5)	0.98
Daily smokers	23 (3.8)	8 (2.8)	15 (5.0)	0.40
Citizenship Norwegian Danish Polish Swedish German English From Asia Africa America Europe Australia Missing	514 6 4 3 3 3 2 3 2 16 2 31	262 2 1 2 3 1 1 1 1 5 1 15	252 4 3 1 0 2 1 2 1 11 1 16	0.19

SD; standard deviation

IQR; interquartile range

BMI; body mass index

NOK; Norwegian kroners

**Table 2:** Physical activity level measured in MET-minutes/week and proportion of women meeting the recommended physical activity level at four measure points for the total sample, by randomization.

Outcome	Time point	Intervention group (n = 295)^c^	*p*^¶^	Control group (n = 295)^d^	*p*^¶^	*p*^¶¶^
TPA^a^	Inclusion	960 (495,1786)		1046 (431,1892)		0.58
	36 weeks	1164 (598,1920)	0.11	720 (308,1594)	**0.002**	**0.001**
	6 months pp	1600 (855,2302)	**<0.001**	1584 (998,2573)	**<0.001**	0.68
	12 months pp	1234 (644,2196)	**0.006**	1253 (675,2265)	**0.006**	0.96
Walking^a^	Inclusion	462 (165,792)		396 (165,800)		0.68
	36 weeks	462 (198,924)	0.63	396 (99,891)	0.52	0.38
	6 months pp	693 (396,1386)	**<0.001**	924 (396,1452)	**<0.001**	0.55
	12 months pp	495 (264,924)	0.062	495 (231,1155)	0.058	0.96
MPA^a^	Inclusion	240 (0,480)		220 (0,480)		0.75
	36 weeks	360 (80,600)	**0.003**	120 (0,480)	0.33	**0.005**
	6 months pp	246 (0,600)	0.092	240 (0,480)	0.33	0.59
	12 months pp	240 (0,480)	0.19	240 (0,480)	0.32	0.65
VPA^a^	Inclusion	0 (0,480)		0 (0,480)		0.49
	36 weeks	0 (0,480)	0.76	0 (0,0)	**<0.001**	**0.003**
	6 months pp	160 (0,800)	**0.009**	240 (0,800)	**0.001**	0.48
	12 months pp	0 (0,780)	0.076	240 (0,720)	**0.011**	0.45
						***p***^**¶¶ef**^
TPA Adherent^e^	Inclusion	1013 (636,1760)				0.24
	36 weeks	1222 (803,1893)	0.11			**0.002**
	6 months pp	1617 (1027,2524)	**<0.001**			0.62
	12 months pp	1013 (551,2013)	0.58			0.19
TPA Nonadherent^f^	Inclusion	975 (431,1905)				0.64
	36 weeks	931 (340,2039)	0.60			**0.023**
	6 months pp	1649 (833,2398)	**0.001**			0.85
	12 months pp	1462 (746,2349)	**0.014**			0.24
				***p*** ^**¶¶¶**^	***p*** ^**¶¶¶**^	***p*** ^**¶¶¶¶**^
Meeting PA recommendations^b^ TPA	Inclusion	72		65		0.067
	36 weeks	75	0.43	56	**0.032**	**<0.001**
	6 months pp	84	**0.001**	88	**<0.001**	0.21
	12 months pp	78	0.13	78	**0.002**	1.000

^a^Values are median with 1^st^ and 3^rd^ interquartile range

^b^Values in percent (%)

Answered IPAQ-SF and included in the analysis

^c^intervention group: inclusion (295), gestational week 36 (255), 6 months postpartum (pp) (239), 12 months pp (212)

^d^control group: inclusion (294), gestational week 36 (254), 6 months pp (231), 12 months pp (212)

^e^Adherent; Intervention group participant attending ≥ 15 (median score) group-exercises

^f^Nonadherent; Intervention group participant attending < 15 (median score) group-exercises

TPA; total physical activity; walking + moderate and vigorous physical activity

MPA; moderate intensity physical activity

VPA; vigorous intensity physical activity

PA; physical activity

¶ Change in PA level compared to time at inclusion (Wilcoxon Signed Ranked Test for TPA, MPA and VPA)

¶¶ Difference in PA level compared to time at inclusion between intervention- and control group (Mann-Whitney U for TPA, MPA and VPA)

**¶¶**^**ef**^ Difference in PA level compared to time at inclusion, between intervention-adherent/intervention-nonadherent and control group (Mann-Whitney U Test for TPA)

¶¶¶ Change in proportions meeting physical activity recommendations of 600 MET-minutes/week compared to time of inclusion (MedCalc for Windows)

^¶¶¶¶^ Difference between intervention- and control group in proportions meeting physical activity recommendations of 600 MET-minutes/week (MedCalc for Windows)

**Table 3:** Physical activity level measured in MET-minutes/week and proportion of women meeting the recommended physical activity level at four measure points, for the physically inactive and the physically active participants at inclusion, by randomization.

		Physically inactive (n = 187)					Physically active (n = 402)				
Outcome	Time point	Intervention group^e^	*p* ^¶^	Control group^f^	*p* ^¶^	*p* ^¶¶^	Intervention group^g^	*p* ^¶^	Control group^h^	*p* ^¶^	*p* ^¶¶^
TPA^a^	Inclusion	332 (70,452)		306 (120,462)		0.99	1333 (892,2316)		1546 (1095,2376)		0.087
	36 weeks	608 (198,1322)	**<0.001**	431 (141,824)	**<0.001**	0.06	1394 (831,2222)	0.31	890 (428,1826)	**<0.001**	**<0.001**
	6 months pp	1554 (799,2235)	**<0.001**	1637 (938,2546)	**<0.001**	0.63	1619 (855,2483)	0.34	1582 (1013,2586)	0.54	0.73
	12 months pp	1144 (605,2194)	**<0.001**	1328 (645,2659)	**<0.001**	0.38	1350 (690,2196)	0.58	1214 (678,2126)	**0.025**	0.25
Walking^a^	Inclusion	116 (0,264)		132 (0,297)		0.61	660 (396,1155)		660 (396,1155)		0.54
	36 weeks	215 (33,462)	**0.001**	248 (0,495)	**<0.001**	0.81	594 (264,1040)	0.34	495 (190,924)	**0.005**	0.10
	6 months pp	693 (462,1172)	**<0.001**	924 (462,1477)	**<0.001**	0.52	792 (396,1386)	0.12	924 (396,1386)	0.14	0.93
	12 months pp	396 (132,792)	**<0.001**	495 (264,1386	**<0.001**	0.12	528 (297,990)	0.71	495 (206,990)	**0.029**	0.16
MPA^a^	Inclusion	0 (0,160)		0 (0,160)		0.94	320 (120,640)		320 (120,640)		0.70
	36 weeks	240 (0,480)	**<0.001**	60 (0,240)	**0.029**	**0.042**	400 (160,720)	0.33	200 (0,480)	**0.024**	**0.019**
	6 months pp	240 (0,560)	**<0.001**	240 (0,720)	**<0.001**	0.96	240 (0,610)	0.35	200 (0,480)	**0.009**	0.29
	12 months pp	240 (0,480)	**<0.001**	240 (10,590	**<0.001**	0.65	240 (0,480)	0.44	240 (0,480)	**0.012**	0.21
VPA^a^	Inclusion	0 (0,0)		0 (0,0)		0.61	160 (0,720)		160 (0,720)		0.89
	36 weeks	0 (0,260)	**<0.001**	0 (0,0)	0.57	**0.021**	0 (0,640)	0.063	0 (0,0)	**<0.001**	**0.008**
	6 months pp	160 (0,800)	**<0.001**	160 (0,720)	**<0.001**	0.92	200 (0,740)	0.96	320 (0,960)	0.63	0.63
	12 months pp	240 (0,880)	**<0.001**	0 (0,720)	**<0.001**	0.79	0 (0,720)	0.48	240 (0,780)	0.94	0.61
TPA Adherent^c^						***p*** ^**¶¶cd**^					***p*** ^**¶¶cd**^
	Inclusion	396 (264,480)				0.17	1353 (863,2085)				**0.038**
	36 weeks	837 (498,1564)	**<0.001**			**0.017**	1388 (900,2117)	0.84			**<0.001**
	6 months pp	1502 (530,2113)	**<0.001**			0.18	1651 (1092,2619)	**0.046**			0.19
	12 months pp	1097 (552,1981)	**0.001**			0.31	937 (550,2040)	0.27			0.57
TPA Nonadherent^d^	Inclusion	223 (0,438)				0.34	1386 (983,2711)				0.48
	36 weeks	389 (80,1060)	**<0.001**			0.41	1408 (621,2536)	0.17			0.051
	6 months pp	1649 (1081,2788)	**<0.001**			0.82	1636 (693,2274)	0.63			0.40
	12 months pp	1189 (609,2460)	**<0.001**			0.62	1554 (810,2324)	0.73			0.17
			***p***^**¶¶¶**^		***p***^**¶¶¶**^	***p***^**¶¶¶¶**^		***p***^**¶¶¶**^		***p***^**¶¶¶**^	***p***^**¶¶¶¶**^
Meeting PA recommendations^b^ TPA	Inclusion	0		0			100		100		
	36 weeks	50	**<0.001**	39	**<0.001**	0.16	85	**<0.001**	66	**<0.001**	**<0.001**
	6 months pp	82	**<0.001**	88	**<0.001**	0.29	84	**<0.001**	88	**<0.001**	0.31
	12 months pp	75	**<0.001**	80	**<0.001**	0.49	79	**<0.001**	76	**<0.001**	0.54

^a^Values are median with 1^st^ and 3^rd^ interquartile range

^b^Values in percent (%)

^c^Adherent; Intervention group participant attending ≥ 15 (median score) group-exercises

^d^Nonadherent; Intervention group participant attending < 15 (median score) group-exercises

Answered IPAQ-SF and included in the analysis Inactive

^e^intervention group: inclusion (84), gestational week 36 (74), 6 months postpartum (pp) (73), 12 months pp (61)

^f^control group: inclusion (103), gestational week 36 (88), 6 months pp (84), 12 months pp (76)

Active

^g^intervention group: inclusion (211), gestational week 36 (181), 6 months postpartum (pp) (166), 12 months pp (151)

^h^control group: inclusion (191), gestational week 36 (166), 6 months pp (147), 12 months pp (136)

TPA; total physical activity; walking + moderate and vigorous physical activity

MPA; moderate intensity physical activity

VPA; vigorous intensity physical activity

PA; physical activity

PP; postpartum

¶ Change in PA level compared to time at inclusion (Wilcoxon Signed Ranked Test for TPA)

¶¶Difference in PA level compared to time at inclusion, between intervention-and control group (Mann-Whitney U Test for total PA)

¶¶^**cd**^ Difference in PA level compared to time at inclusion, between intervention-adherent/intervention-nonadherent-and control group (Mann-Whitney U Test for TPA)

¶¶¶ Change in proportions meeting PA recommendations of 600 MET-minutes/week compared to time of inclusion (MedCalc for Windows)

¶¶¶¶ Difference between intervention- and control group in proportions meeting PA recommendations of 600 MET-minutes/week (MedCalc for Windows)

**Table 4:** Physical activity level measured in MET-minutes/week and proportion of women meeting the recommended physical activity level at four measure points, for the normal weight and the overweight/obese participants, by randomization.

		Normal weight (n = 423)					Overweight or obese (n = 166)					Comparing BMI categories	
Outcome	Time point	Intervention group^c^	*p* ^¶^	Control group^d^	*p* ^¶^	*p* ^¶¶^	Intervention group^e^	*p* ^¶^	Control group^f^	*p* ^¶^	*p*^¶¶^	*p* ^¶¶^	
												IG	CG
TPA^a^	Inclusion	990 (480,1764)		1100 (443,1847)		0.85	924 (613,1835)		921 (399,1923)		0.61	0.83*	
	36 weeks	1200 (659,2013)	0.077	777 (375,1631)	**0.014**	**0.003**	990 (480,1762)	0.86	500 (153,1388)	0.057	0.069	0.41	0.31
	6 months pp	1649 (998,2451)	**<0.001**	1583 (1009,2563)	**<0.001**	1.00	1268 (690,2285)	**0.029**	1620 (875,2678)	**0.004**	0.46	0.88	0.37
	12 months pp	1195 (633,2169)	0.15	1328 (635,2234)	**0.043**	0.67	1435 (716,2490)	**0.004**	1136 (701,2405)	**0.036**	0.65	0.43	0.12
Walking^a^	Inclusion	462 (132,792)		396 (165,792)		0.77	495 (264,1139)		396 (198,899)		0.25	0.20*	
	36 weeks	462 (198,924)	0.29	396 (99,924)	0.81	0.33	429 (140,916)	0.55	347 (99,693)	0.39	0.74	0.28	0.57
	6 months pp	792 (462,1386)	**<0.001**	924 (396,1592)	**<0.001**	0.97	660 (297,1386)	0.13	693 (462,1287)	**0.005**	0.29	0.089	0.67
	12 months pp	495 (264,924)	0.074	512 (239,1155)	0.12	0.92	495 (248,990)	0.51	462 (198,1155)	0.24	0.77	0.74	0.89
MPA^a^	Inclusion	240 (40,480)		240 (0,480)		0.41	240 (0,480)		160 (0,420)		0.28	0.19*	
	36 weeks	360 (120,600)	**0.030**	160 (0,480)	0.58	**0.046**	320 (0,480)	**0.033**	20 (0,480)	0.28	**0.034**	0.61	0.78
	6 months pp	240 (0,720)	0.10	240 (0,480)	0.41	0.50	240 (0,480)	0.64	120 (0,480)	0.62	0.88	0.60	0.89
	12 months pp	240 (0,480)	0.78	240 (0,480)	0.99	0.65	320 (0,480)	**0.042**	260 (120,630)	**0.023**	0.84	0.15	0.047
VPA^a^	Inclusion	0 (0,480)		0 (0,480)		0.24	0 (0,460)		0 (0,420)		0.83	0.41*	
	36 weeks	0 (0,640)	0.82	0 (0,0)	**<0.001**	**0.005**	0 (0,480)	0.82	0 (0,0)	0.095	0.44	0.99	0.11
	6 months pp	240 (0,800)	0.13	240 (0,800)	**0.010**	0.53	80 (0,640)	**0.007**	0 (0,960)	**0.028**	0.50	0.32	0.26
	12 months pp	0 (0,660)	0.73	200 (0,720)	**0.036**	0.19	320 (0,840)	**0.004**	280 (0,720)	0.16	0.58	0.058	0.80
						***p*** ^**¶¶gh**^					***p*** ^**¶¶gh**^		
TPA Adherent^g^	Inclusion	1164 (636,1840)				0.16	891 (630,1396)				0.93		
	36 weeks	1238 (876,2013)	0.32			**0.031**	1158 (669,1782)		0.17		**0.017**		
	6 months pp	1617 (1019,2362)	**0.009**			0.48	1612 (1022,2837)		**0.009**		0.86		
	12 months pp	1004 (533,2384)	0.61			0.10	1107 (602,2170)		0.062		0.94		
TPA Nonadherent^h^	Inclusion	975 (400,1804)				0.29	975 (545,2078)				0.47		
	36 weeks	1082 (405,2166)	0.13			**0.007**	723 (198,1748)		0.29		0.54		
	6 months pp	1743 (1002,2482)	**<0.001**			0.50	1155 (688,2396)		0.48		0.19		
	12 months pp	1455 (693,2373)	**0.026**			0.36	1508 (787,2384)		0.029		0.52		
			***p*¶¶¶**		***p*¶¶¶**	***p***¶¶¶¶		***p*¶¶¶**		***p*¶¶¶**	***p*****¶**¶¶¶		
Meeting PA recommendations^b^ TPA	Inclusion	69		65		0.38	76		64		0.093		
	36 weeks	78	0.049	59	0.21	**<0.001**	69	0.31	48	0.062	**0.012**		
	6 months pp	85	**<0.001**	89	**<0.001**	0.27	80	0.54	83	**0.019**	0.67		
	12 months pp	77	0.097	76	**0.021**	0.84	82	0.38	80	**0.012**	0.68		

^a^Values are median with 1^st^ and 3^rd^ interquartile range

^b^Values in percent (%)

^g^Adherent; Intervention group participant attending ≥ 15 (median score) group-exercises

^h^Nonadherent; Intervention group participant attending < 15 (median score) group-exercises

TPA; total physical activity; walking + moderate and vigorous physical activity

MPA; moderate intensity physical activity

VPA; vigorous intensity physical activity

PA; physical activity

pp; postpartum

Answered IPAQ-SF and included in the analysis

Normal weight

^c^intervention group: inclusion (203), gestational week 36 (175), 6 months postpartum (pp) (168), 12 months pp (150)

^d^control group: inclusion (220), gestational week 36 (192), 6 months pp (178), 12 months pp (164)

Overweight/obese

^e^intervention group: inclusion (92), gestational week 36 (80), 6 pp (71), 12 months pp (62)

^f^control group: inclusion (74), gestational week 36 (62), 6 months pp (53), 12 months pp (48)

¶ Change in PA level compared to time at inclusion /proportion meeting PA recommendations compared to time at inclusion (Wilcoxon Signed Ranked Test)

¶¶Difference in PA level compared to time at inclusion between intervention-and control group (Mann-Whitney U Test)

**¶¶**^**gh**^ Difference in PA level compared to time at inclusion, between intervention-adherent/intervention-nonadherent-and control group (Mann-Whitney U Test for TPA)

*Difference in physical activity level at inclusion, between normal weight and overweight/obese participants (Mann-Whitney U Test for TPA)

¶¶¶ Change in proportions meeting PA recommendations of 600 MET-minutes/week compared to time of inclusion for TPA (MedCalc for Windows)

¶¶¶¶ Difference between intervention- and control group in proportions meeting PA recommendations of 600 MET-minutes/week for TPA (MedCalc for Windows)

Among the intervention group participants answering IPAQ-SF at inclusion, 269 out of 295 (91.2%) attended at least one exercise class. The number of classes attended varied between 0 and 38, with a median of 15 (IQR 7,23).

The IPAQ-SF response rate was 86%, 80% and 72% at gestational week 36, six and 12 months postpartum, respectively. Compared to participants answering the questionnaires, the non-responders differed in some baseline characteristics at the different time points. Non-responders at gestational week 36 were characterized by a larger proportion of smokers (8% versus 3%, *p* = 0.049), they were younger (26.7 years (SD 4.6) versus 28.2 years (SD 4.3), *p* = 0.003) and participated in fewer group-exercise classes (7.7 (SD 6.8) versus 16.2 (SD 10.2), *p*<0.001) compared to responders. At 12 months postpartum fewer non-responders had higher educational level (29% versus 38%, *p* = 0.030).

### Total sample, by randomization

**Fig 2:**
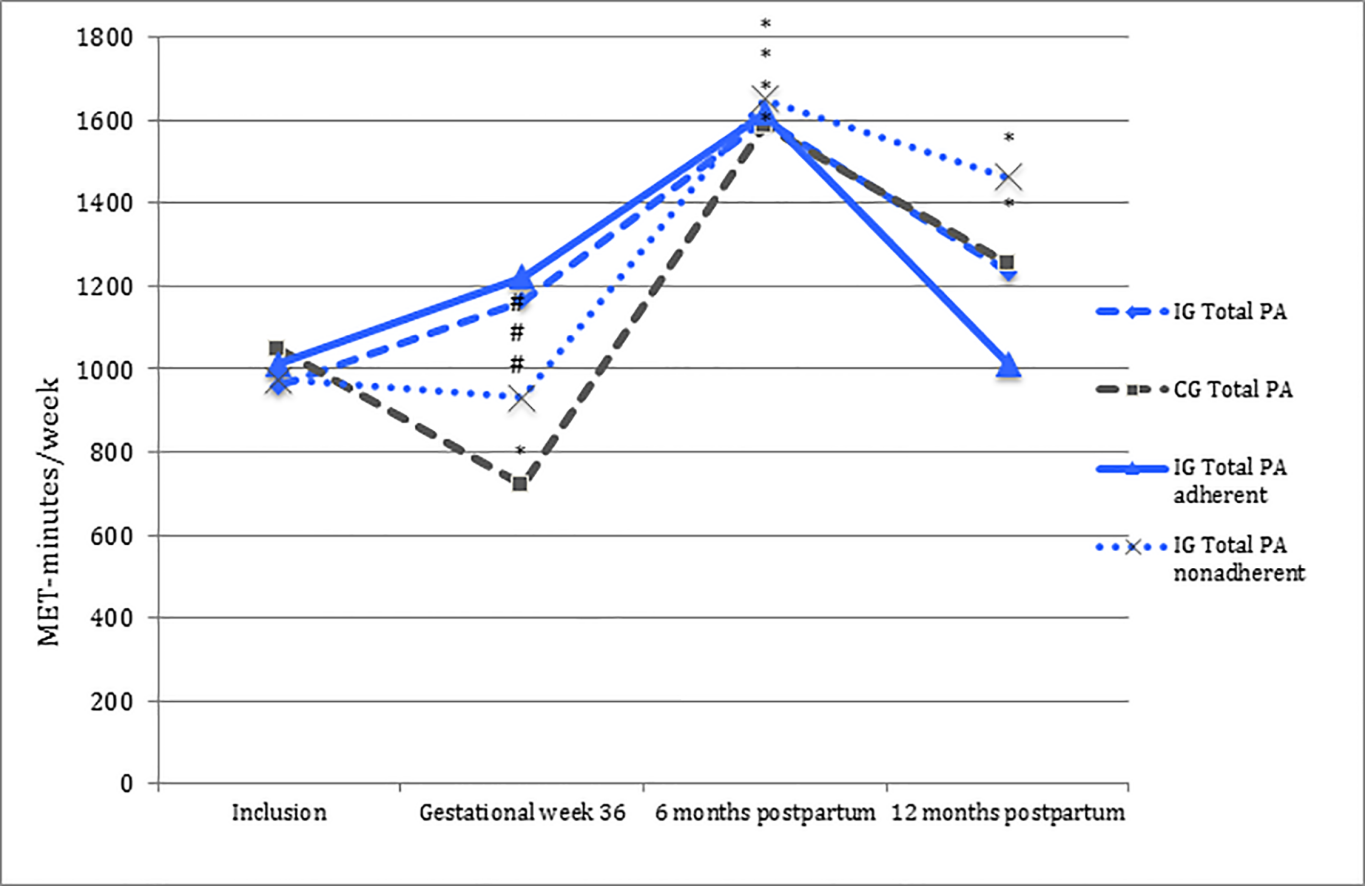
Total physical activity level in MET-minutes/week. IG; intervention group (n = 295). CG; control group (n = 294). Total PA; total physical activity; walk + moderate physical activity + vigorous physical activity. - - - blue = intervention groups and - - - dark grey = control group. ^**_______**^blue = intervention group adherent and ^….^blue = intervention group nonadherent. *Statistically significant change in physical activity level compared to time of inclusion (*p*<0.05). # Statistically significant change in total PA level compared to time of inclusion, between intervention group, intervention adherent/nonadherent subgroups and control group (*p*<0.05).

At gestational week 36, there was a significant difference between the intervention and the control group in level of total physical activity, MPA and VPA, but not for walking (Table 2). The intervention group maintained total physical activity level (+204 MET-min/week, *p* = 0.11), while the control group decreased total physical activity level (-326 MET-min/week, *p* = 0.002) at gestational week 36 compared to time of inclusion (Table 2, Fig 2). Both the intervention- and the control group maintained time spent walking at gestational week 36 compared to time of inclusion (Table 2).

In the postpartum period, no significant differences were observed between the intervention and the control group in physical activity level. Both groups increased their total physical activity level at six months and 12 months postpartum compared to time of inclusion (Table 2, Fig 2).

Analyzing intervention group participants classified as adherent or nonadherent to the group-exercise intervention did not alter the positive intervention-effect seen at gestational week 36 compared to time of inclusion (Table 2, Fig 2). Both the adherent (+209 MET-min/week, *p* = 0.11) and the nonadherent intervention participants (-44 MET-min/week, *p* = 0.60) maintained total physical activity level at gestational week 36 compared to time of inclusion, in contrast to the control group (-326 MET-min/week, *p* = 0.002). Adherent and nonadherent intervention participants had similar baseline characteristics except for mean gestational age at inclusion (15.6 weeks (SD 2.3) versus 16.5 weeks (SD 2.6, respectively *p* = 0.009), as well as a larger proportion amongst the adherent with a higher educational level (≥4 years of college/university, 40.0% versus 28.4%, *p* = 0.040).

The mixed between-within subjects ANOVA showed that group differences varied significantly across time points in terms of total physical activity level; Wilks´ Lambda = 0.94, F (3,300) = 6.04, *p* = 0.001, partial eta.squared = 0.057. The main effect comparing the two groups of randomization over time was not significant; F (1,302) = 0.36, *p* = 0.55, partial eta.squared = 0.002. Results remained unchanged in adjusted analyses.

The proportion of women meeting physical activity recommendations based on total physical activity level was significantly higher in the intervention group than the control group at gestational week 36, while there were no differences between groups at time of inclusion or in the postpartum period (Table 2).

### Physically inactive versus physically active

**Fig 3:**
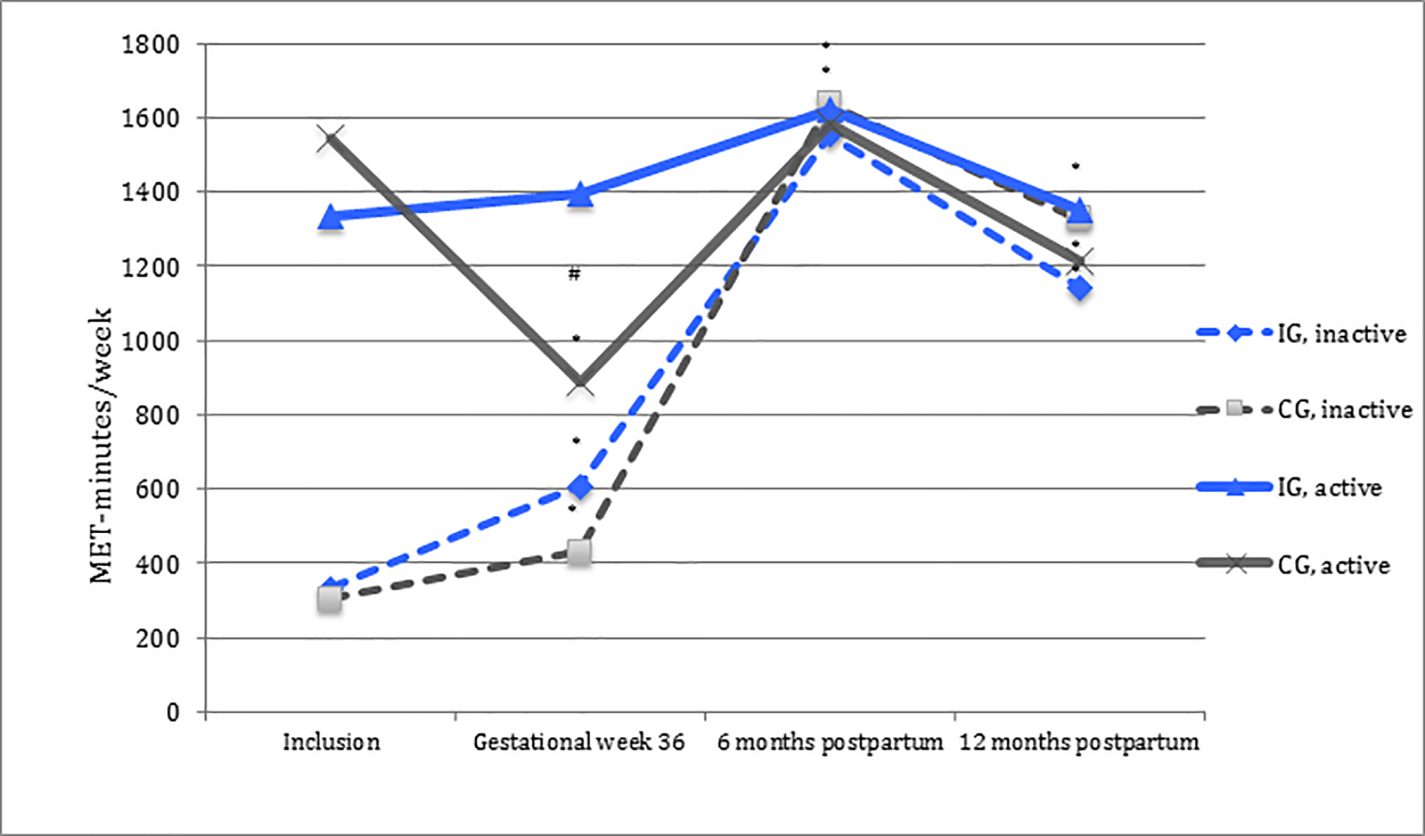
Total physical activity level in MET-minutes/week for the inactive and the active participants. - - - physically inactive and ^**_______**^ physically active, blue = intervention group (IG) and dark grey = control group (CG). *Significant change in physical activity level compared to time of inclusion (*p*<0.05). #Significant difference in physical activity level between the intervention group and the control group compared to time of inclusion (*p*<0.05).

When analyzing the participants according to whether they were physically inactive or active at time of inclusion, a difference in level of total physical activity (*p*<0.001), MPA (*p* = 0.019) and VPA (*p* = 0.008) between the intervention and the control group was shown among the physically active participants (n = 402) at gestational week 36, but not in the postpartum period, compared to time of inclusion. The active intervention group participants maintained their level of total physical activity, walking, MPA and VPA at gestational week 36 compared to time of inclusion, while the active control group participants correspondingly showed a significant decline in their physical activity level (Table 3, Fig 3).

Among physically inactive participants (n = 187), a difference in MPA and VPA level was shown between the intervention and the control group at gestational week 36 compared to time of inclusion. No significant difference in total physical activity level was observed at any of the measure time-points. Irrespective of randomization group, the inactive participants significantly increased their level of total physical activity, walking and MPA at gestational week 36, as well as in the postpartum period, compared to time of inclusion (Table 3, Fig 3).

**Fig 4:**
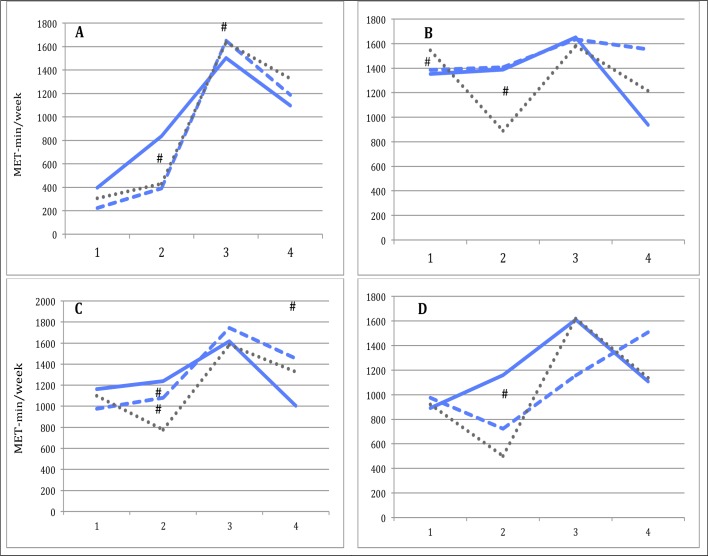
Total physical activity level measured between participants in the intervention group adherent and nonadherent to group-exercises and the control group. 1: time of inclusion, 2: gestational week 36, 3: 6 months postpartum, 4: 12 months postpartum. ^_____^Blue; intervention adherent. -----Blue; intervention nonadherent. .....Dark grey; control group. A; Inactive; participants physically inactive at time of inclusion (<600 MET-min/week of MVPA). B; Active; participants physically active at time of inclusion (≥600 MET-min/week of MVPA). C; Normal weight; prepregnancy BMI < 25 kg/m^2^. D; Overweight/obese; prepregnancy BMI ≥ 25 kg/m^2^. **#** Statistically significant change in total physical activity level compared to time of inclusion, between intervention group adherent subgroup and control group (*p*<0.05).

The number of exercise classes attended varied, with a median of 12 (IQR 3,21) for physically inactive participants and a median of 17 (IQR 8,24) for physically active participants. Adherence to exercise classes influenced intervention-effect at gestational week 36 for both inactive and active participants (Table 3, Fig 4).

There was no difference in proportions meeting physical activity recommendations between the intervention group and the control group at gestational week 36 or in the postpartum period among the inactive participants. Among the active participants, a higher proportion met the physical activity recommendations at gestational week 36 in the intervention group compared to the control group, while there was no difference between randomization groups in the in the postpartum period (Table 3).

### Body mass index categories

**Fig 5:**
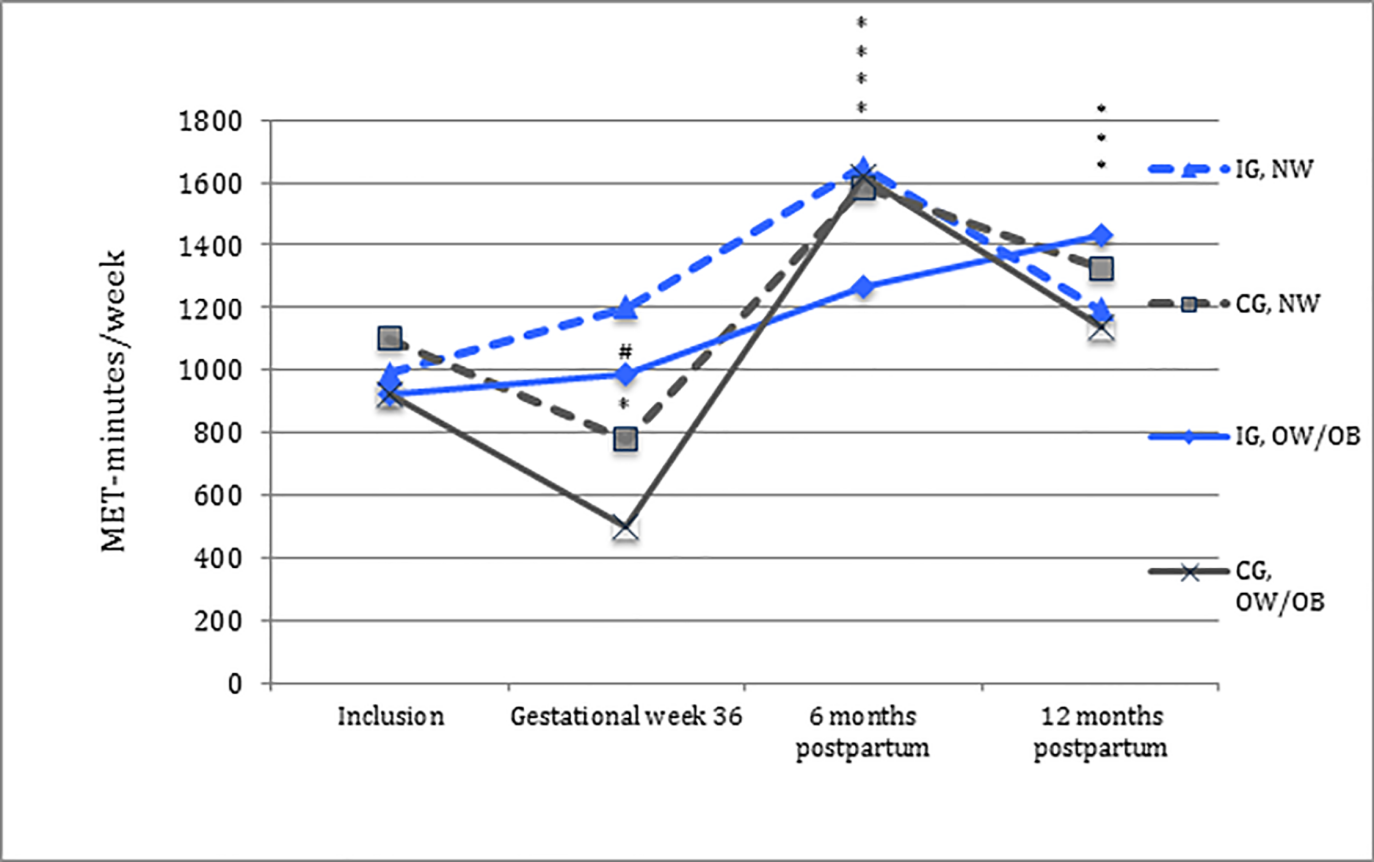
Total physical activity level in MET-minutes/week for the normal weight and the overweight/obese. IG; intervention group. CG; control group. NW; normal weight. OW/OB; overweight/obese. - - - normal weight and ^**_______**^ overweight/obese, blue = intervention group (IG) and dark grey = control group (CG). *Significant change in physical activity level compared to time of inclusion (*p*<0.05). #Significant difference in physical activity level between the intervention group and the control group compared to time of inclusion (*p*<0.05).

When differentiating participants according to pre-pregnancy BMI (see Table 4), a significant difference in total physical activity level between the intervention and the control group was observed at gestational week 36 for the normal weight subgroup, but not for the overweight/obese subgroup. In both BMI subgroups, MPA level was significantly higher at gestational week 36 compared to time of inclusion among intervention-group participants, and significantly higher compared to corresponding control group participants; time spent walking was maintained for both intervention and control group participants. Postpartum, no difference in total physical activity level between the intervention and the control groups was reported in the normal weight or the overweight/obese subgroups, compared to time of inclusion (Table 4, Fig 5).

Change in physical activity level was examined with respect to groups of randomization and possible differences between BMI categories; the normal weight and overweight/obese subgroups had a similar change in physical activity level in pregnancy. Physical activity level at specific intensities for BMI categories is shown in Table 4.

The number of classes attended varied with a median of 15 (IQR 7,23) for normal weight participants and a median of 14 (IQR 6,22.5) for overweight/obese participants. Adherence to exercise classes did not influence the positive intervention-effect on physical activity level at gestational week 36 among the normal weight participants. In the overweight/obese subgroup, intervention-effect at gestational week 36 was only observed among the adherent participants (Table 4, Fig 4).

The proportion of women meeting the physical activity recommendations was significantly greater in the intervention group than the control group at gestational week 36, in both normal weight (*p*<0.001) and overweight/obese (*p* = 0.012) subgroups, while there were no differences between randomization groups in the postpartum period (Table 4).

## Discussion

In the present study, we found that pregnant women receiving a combined lifestyle intervention including supervised exercise classes maintained total physical activity level in late pregnancy, in contrast to women receiving routine prenatal care who reported a decrease in their physical activity level. Also, a greater proportion of women receiving the intervention met physical activity recommendations in late pregnancy compared to women receiving routine prenatal care. However, a long-term effect of the intervention was not observed in the postpartum period.

Of note, the intervention’s effect on physical activity levels in late pregnancy differed depending on physical activity level at trial inclusion and pre-pregnancy BMI category; intervention effect was only observed among participants who were physically active at inclusion or entered the pregnancy as normal weight. Adherence to exercise-classes seemed, however, to give a positive intervention effect in the physically inactive and overweight/obese subgroups as well.

### Total sample, by randomization

At gestational week 36, the reported difference in total physical activity level between the intervention group and the control group was 444 MET-min/week, equivalent to 111 minutes of physical activity of moderate intensity. This constitutes over two-thirds of recommended minimum weekly physical activity, and presumably provided the intervention group participants with additional health benefits. The NFFD intervention seemed to prevent the downward trend in total physical activity seen among the control group participants and reported by previous studies [17–19]. Also, in late pregnancy, 75% of the women in the intervention group met the physical activity recommendations based on total physical activity level, a considerably larger proportion of women compared to the control group (56%). Proportions meeting the recommended physical activity level in the NFFD trial are in line with self-reported levels of total physical activity in the general female population in Norway (63% - 68%, aged 20–49 years) [16].

Adherence to exercise-classes did not alter the positive intervention-effect of the NFFD intervention on total physical activity level in late pregnancy. The positive intervention-effect of a combined lifestyle intervention on physical activity level seen in late pregnancy in our study is comparable with results from several previous studies, comprising both combined lifestyle interventions [10, 28, 29, 31, 43] and physical activity interventions alone [12, 32, 34]. In contrast, a study of a combined lifestyle modification with focus on walking reported a positive intervention-effect, but nonetheless reported a decrease in physical activity level in late pregnancy among intervention group participants [30]. Further, in two studies of physical activity interventions alone, with design similar to ours [33] or with higher frequency but similar intensity [5], no intervention-effect on physical activity level in pregnancy was demonstrated. In our study, level of walking remained unchanged from early to late pregnancy, both for the intervention and the control group. This is in line with previous observational studies, where walking was the most commonly reported mode of exercise in pregnancy [18, 19]. The lack of intervention effect on walking found in our study may partly be explained by the NFFD physical activity intervention, which focused on aerobic and strength exercises held in exercise classes twice weekly. However, walking might have been expected to increase in the intervention group compared to the control group, as the intervention group was motivated to take part in 30 minutes of cardio exercise three times/week outside the group exercise classes. On the other hand, a lower reported level of walking in late pregnancy among intervention group participants may have several explanations. Time spent going to exercise classes might create scheduling constraints, and class attendance might remove the perceived need for additional recreational walking/exercise.

Several of the previous intervention studies were conducted in selective pregnancy populations, such as overweight or obese women [5, 10, 28, 30, 34], women at increased risk of developing gestational diabetes [32] or women who were physically inactive prior to pregnancy [12, 33], making comparison challenging. Furthermore, variations in reporting of physical activity level, such as physical activity units or score [29, 31, 43], fitness score [10], cardiorespiratory- or aerobic fitness [12, 33, 34] or, as in our study, physical activity intensity/MET-minutes [28, 30], complicates comparison even more. The challenge of comparing studies is amplified by heterogeneous measurement methods, differing frequency, intensity and duration of exercise implements, as well as variation in timing of and adherence to the exercise interventions.

Beyond the group exercises in our study, participants in the intervention group were encouraged to exercise for at least additional 30 minutes of MPA at least three times per week. The importance, benefits and safety of physical activity in pregnancy was also reinforced through the NFFD pamphlet and web-site, and the social support obtained by meeting other intervention participants. All these factors may have contributed to the maintenance of physical activity level through pregnancy for intervention participants in this study.

During the first year postpartum, no long-term effect of the NFFD intervention was observed; both the intervention and the control group participants increased physical activity level compared to time of inclusion, with the highest peak after six months, where walking and VPA seemed to be the strongest contributors. The observed drop in physical activity level between early and late pregnancy in the control group, as well as the increased physical activity level postpartum, correspond well with previous observational studies of pregnant women based on sensory-based physical activity data [17] and self-reported physical activity data [44]. In the present study, access to group exercise classes ceased after delivery, and might be a reason why the intervention-effect was only seen in pregnancy and not in the postpartum period. Also, paid maternity leave in Norway typically lasts for 10 months, during which time it is common for new mothers to gather in local maternity groups. These meetings often involve walks with baby strollers and likely include discussions of lifestyle habits. This kind of socializing between participants in the intervention and the control group may partly explain the lack of intervention-effect in the postpartum period. Further, most women in Norway are still on maternity leave at six months postpartum, which might explain why the physical activity level was higher at this point compared to 12 months postpartum, where most of the women have returned to their daily work or studies.

There are few comparable intervention trials with follow-up in the postpartum period. Similar to our study, the Danish LiP-study involving a combined lifestyle intervention for obese women in pregnancy, reported no difference in physical activity level or physical fitness between the intervention and the control group participants at six months postpartum [10]. Further, Dodd et al. reported no intervention-effect four months postpartum compared to time of inclusion in an Australian study including over 2000 overweight or obese women, a study primarily of diet and exercise advice, although a small nested subgroup received the option of joining a walking group [30].

### Physical activity categories

Further, no other studies, to the authors’ knowledge, have investigated the effect of a lifestyle intervention stratified according to participant physical activity level at inclusion. This approach revealed that the intervention-effect differed between the physically inactive versus the active participants, suggesting that a “one-size fits all” intervention program may not be sufficient. On the other hand, physical activity level reported at inclusion might be influenced by pregnancy complaints such as nausea or fatigue, which may lead to participants being miscategorized. Optimally, classification of physical activity level should take place prior to pregnancy. The intervention group participants who were physically active at time of inclusion in our study had, compared to the control group, a significantly higher physical activity level in late pregnancy. Physically inactive participants at trial inclusion, on the other hand, were dependent on exercise-adherence to achieve intervention-effect, which corresponds well with the positive intervention effect seen in a physical activity intervention study reported by Price et al., including pregnant women in the US who were physically inactive prior to pregnancy, where 77% of the participants were adherent to exercise-classes [12]. In contrast, Halvorsen et al. found no intervention-effect on cardiorespiratory fitness when performing a 12 week physical activity intervention for previously physically inactive women in Norway, where mean adherence to exercise-classes were 83% [33]. Comparison of the NFFD study to these two studies must be done with caution, however, as both Price and Halvorsen report change in aerobic fitness or cardiorespiratory fitness, and not physical activity level as in our study.

### Body mass index categories

Positive intervention effect on total physical activity level in late pregnancy was only demonstrated for women with normal weight in our study. Overweight/obese women maintained their total physical activity level, but were dependent on exercise-class adherence to demonstrate a significant difference from the control group participants. Very few studies are available for comparison, as results are rarely stratified according to participants’ BMI category. In a Canadian study with similar intervention components as the NFFD trial [29], no intervention-effect on physical activity level was demonstrated among the overweight/obese, only among normal-weight participants. As maternal obesity is a risk factor for adverse pregnancy outcomes such as excessive gestational weight gain and gestational diabetes, these women would especially benefit from being physically active in pregnancy [6]. In future studies, it would be of interest to thoroughly explore the possible differences between adherent and non-adherent participants, for example through qualitative methods such as interviewing.

Exercise classes were at fixed time points in the NFFD trial, possibly excluding some participants for practical reasons [45]. More flexible timetables for exercise classes and increased accessibility with public transport may increase adherence in future exercise interventions.

### Strengths and limitations

To the best of our knowledge, the NFFD trial is one of the largest randomized controlled trials of an antenatal combined lifestyle-intervention including supervised exercise classes. The study included both normal-weight and overweight/obese participants and continued follow-up the first year postpartum. Another strength is the high response rate observed throughout the study period, with 86%, 80% and 72% of the participants answering the physical activity questionnaire at gestational week 36, six and 12 months postpartum, respectively. Finally, the supervised group exercises, based on national and ACOG physical activity guidelines [38], contained both aerobic and strength exercises, which is reported to be more favourable for maternal outcomes than using only one exercise modality [46].

We have previously demonstrated that the exercise program in the NFFD trial was feasible, safe and well tolerated in pregnancy, and no participants in the intervention group reported injuries related to the group-exercises [45]. Furthermore, the NFFD intervention did not demonstrate any adverse maternal or neonatal outcomes [9].

Limitations in the present study include reliance on self-reported physical activity level, as self-reported questionnaires have known limitations, including under- or over reporting of physical activity level [42, 47]. Traditionally, self-reported measures of physical activity have been the tool for assessing physical activity in most trials conducted during pregnancy, and no questionnaire seems superior to another [48]. IPAQ-SF has shown low validity both in the general population (correlation coefficient 0.09–0.39) [49] and among pregnant women (correlation coefficient 0.08–0.39) [42], weakening its use as an indicator of a person’s physical activity level at a given time point. Use of an objective measure of physical activity level, such as an accelerometer, would have strengthened the study quality. Nevertheless, we have four repeated measures with the same questionnaire, IPAQ-SF, which has shown satisfactory test-retest reliability for both the general population (pooled Spearman rho 0.76) [41] and for use in pregnancy (intraclass correlation coefficient 0.81–0.84) [42]. Thereby one could argue for IPAQ-SF´s value in measuring change in physical activity level at group level over time. The failure to differentiate the amount of physical activity performed during and outside of group exercise classes is another limitation of the present study.

Furthermore, a potential bias in our study may be the relatively high socioeconomic status of participants, which may potentially influence their physical activity level [50]. In addition, participants were predominantly white, of Norwegian decent. These factors may lower the external validity of our finding.

As mentioned, social contact between participants in the intervention- and control groups after giving birth may partly explain the lack of intervention-effect in the postpartum period. In designing the NFFD trial, cluster randomization of healthcare clinics was discussed as an alternative to individual randomization, but we refrained from using this approach due to the other forms of bias that would be introduced, including possible socioeconomic differences between the health care clinics’ catchment areas.

### Perspectives

Investing in preventive measures to improve physical activity habits in pregnancy may positively influence the lifestyle of a new family, be an important contribution to improving public health and restricting future health care costs.

Due to limitations in self-reporting [47], we recommend future research in pregnancy and the postpartum period to include objective assessment of physical activity in addition to subjective measures. Also, future pregnancy interventions should address postpartum physical activity, perhaps extending exercise-intervention beyond delivery, as the intervention-effect appeared to cease after delivery in our study. Further, qualitative studies interviewing postpartum women after participation in a lifestyle intervention during pregnancy could give valuable information on determinants for maintaining physical activity levels after intervention is completed, similar to the study by Miller and Brown [51]. This may aid in planning future interventions and public health initiatives.

The effect of the NFFD lifestyle intervention on physical activity level in late pregnancy was dependent on exercise-adherence in the overweight/obese and physically inactive subgroups, two selected groups at increased risk of pregnancy complications [1, 21]. This should inspire future research to focus on factors influencing adherence to exercise-interventions, as well as early onset interventions, ideally starting pre-pregnancy. Also, the difference in intervention-effect demonstrated in our study based on physical activity level at inclusion, should encourage future studies to offer individualized physical activity interventions based on women’s initial physical activity level.

### Conclusion

The NFFD lifestyle intervention, including twice-weekly supervised exercise classes, succeeded in engaging pregnant women to maintain physical activity level in late pregnancy, a period when the majority of pregnant women experience an overall decrease in physical activity level. The proportion of women meeting the recommended physical activity level in late compared to early pregnancy remained stable among the intervention group participants (72% to 75%), in contrast to the control group, where the proportion of participants meeting recommendations declined from 65% to 56%. Further, adherence to group-exercises had appeared to have an impact on the intervention-effect among both the overweight/obese and physically inactive participants. However, this combined lifestyle intervention failed to demonstrate any long-term effect on physical activity level, as no differences were found between randomization groups in the postpartum period.

## Supporting information

S1 File SPSS-file with all relevant data underlying the findings in the present paper. (SAV)
